# Characterization of *Chlorella sorokiniana*, UTEX 1230

**DOI:** 10.3390/biology7020025

**Published:** 2018-04-13

**Authors:** Alessandro Marco Lizzul, Aitor Lekuona-Amundarain, Saul Purton, Luiza Cintra Campos

**Affiliations:** 1 Unit 12, Ball Mill Top Business Park, Hallow, Worcester WR2 6PD, UK; 2 Department of Environmental Engineering, University College London, Gower Street, London WC1E 6BT, UK; aitor.lekuona.amundarain@gmail.com; 3 Department of Structural and Molecular Biology, University College London, Gower Street, London WC1E 6BT, UK; s.purton@ucl.ac.uk

**Keywords:** *Chlorella sorokiniana*, UTEX1230, productivity, yield, characterization

## Abstract

This paper characterizes the strain *Chlorella sorokiniana* UTEX 1230 within a laboratory setting using a 1 L bubble column. The findings show that productivity can be trebled under mixotrophic conditions (from 0.2 g·L^−1^·d^−1^ to 0.66 g·L^−1^·d^−1^) with the addition of sodium acetate. The results also indicate that both the growth rate and final yield increase with the cultivation temperature, with most parameters showing an optimum in the range of 30–35 °C. The maximum specific growth rate was found to be in the region of 0.12 h^−1^ at a surface irradiance between 100–500 µE·m^−2^·s^−1^. This high growth rate makes the strain particularly suited to the rapid production of biomass, suitable for either whole cell bioprocessing or bioremediation. However, the relatively low lipid productivity (9.2 mg·L^−1^·d^−1^) confirms previous findings which would indicate poor applicability for biodiesel production. The strain shows greater promise in wastewater treatment applications with removal rates of nitrogen and phosphorus in the region of 37 and 30 mg·L^−1^·d^−1^ respectively. Furthermore, the findings show that a fed-batch strategy to inorganic nutrient loading can increase the final yield by around 50% compared to a conventional batch run. This is particularly interesting as fed-batch production techniques are rarely used within microalgal cultivation, so provide an interesting avenue for further investigation. Overall, the findings show that *C. sorokiniana* UTEX 1230 is a robust and fast-growing microalgal strain suitable both for the laboratory and scale-up.

## 1. Introduction

Algae constitute a diverse set of photosynthetic organisms, which can range in size from single cellular bodies to multicellular seaweeds. Extant specimens display polyphyletic evolution and can be found in many kingdoms. Current estimates place the number of algal species between 200,000 and 800,000, of which approximately 35,000 have been classified [1]. The most commonly cultivated microalgal species have a selection of favorable bioprocessing characteristics; including the capacity to produce higher levels of desirable lipids or valuable secondary metabolites [2]. Of the commercially exploited microalgae the *Chlorella* genus is particularly prominent and classified within the *Trebouxiophyceae* family under the division of *Chlorophyta*. They consist of many unicellular sub-species, distributed in both fresh and saline environments. Characteristic features include a smooth cell wall and a non-flagellated, generally spherical morphology; with the size of the various species found to be within a range of 2–10 μm in diameter. To date there are more than 20 characterized *Chlorella* species, with over 100 described strains [3,4]. Members of the species have been reported to have considerable potential for industrial applications; due in part to their relatively rapid and robust growth characteristics.

*Chlorella sorokiniana* is a sub-species first isolated in 1953 by Sorokin, and originally believed to be a thermotolerant mutant of *Chlorella pyrenoidosa* [5,6]. This taxonomic identification was subsequently changed during the late 1980s and early 1990s when chloroplast 16S rDNA and 18S rRNA profiling identified *C. sorokiniana* as a separate species [3,7,8,9]. This sub-species is a small (2–4.5 µm diameter), robust single celled alga that is capable of mixotrophic growth on various carbon and nitrogen sources, making it ideal for cultivation on waste feedstock [10,11]. Previous findings report that optimal growth can be obtained at temperatures between 35–40 °C [12]; with phototrophic doubling times as low as 4–6 h [13]. Growth under mixotrophic and even heterotrophic conditions was observed to be even faster, with a preference for sugars such as glucose or simple organic acids such as acetate [14,15,16].

The species is widely recognized as having industrial potential, and has been shown to be sufficiently robust for scale-up in air-mixed [17] or liquid-mixed photobioreactors [15]. Previous work has also demonstrated that *C. sorokiniana* is able to grow on wastewaters [10,11] under conditions that would be unfavorable for other algal species; including carbon dioxide supplementation from flue gas [18] and higher temperature cultivation [12]. Productivity under these conditions could be expected to be in the region of 0.25–35 g·L^−1^·d^−1^, while nitrogen and orthophosphate levels were reduced by as much as 90% and 70% respectively [19]. Analysis of *C. sorokiniana* dry weight shows that the species is composed on average of 40% protein, 30–38% carbohydrate and 18–22% lipid [20,21,22]. Prior research has shown that *C. sorokiniana* biomass may be well suited to bulk commodity production, in particular the large scale production of lipid for biofuel [23,24]. Some other specific compounds of commercial interest include antioxidants such as carotenoids, which make up to 0.69% of dry weight under extremophilic conditions [25]. Furthermore, research has shown that genetic transformation of *C. sorokiniana* is possible, opening up routes for the expression of a range of transgenic products [26].

The results presented in this paper provide a detailed experimental exploration of the parameter space for *Chlorella sorokiniana*, UTEX 1230 at laboratory scale. The research is benchmarked against literature values to offer insight into the potential for subsequent scale-up. 

## 2. Materials and Methods

### 2.1. Experimental Design

#### 2.1.1. Reactor System

**Figure 1:**
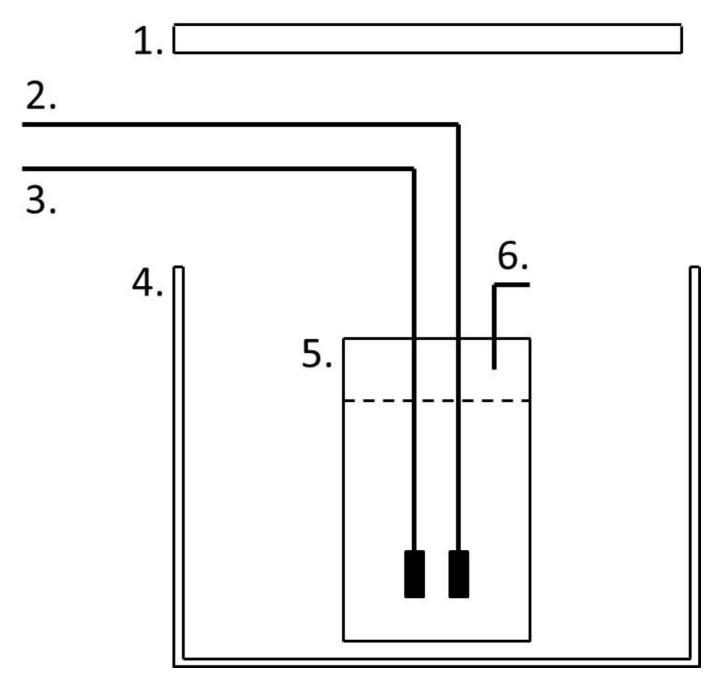
The 1 L Duran bottle reactor. (1) Light source. (2) Mixing airline. (3) Exhaust gas line from compressor. (4) Growth chamber. (5) Culture vessel. (6) Gas and sampling outlet [18].

The reactor system used for the experiments was based on a 1 L Duran^®^ (Camlab, UK) (Figure 1). Mixing within the system was induced by aerating the reactor, and lighting was varied depending on the experiment [18].

#### 2.1.2. Growth with Different Carbon Sources

These experiments were designed to investigate the differences in growth rates under mixotrophic and phototrophic conditions. This was achieved by cultivating *C. sorokiniana* either with or without the addition of 2 g·L^−1^ of sodium acetate within BBM (Sigma, Welwyn Garden City, UK), or with the addition of 5 cm^3^/min of 99.5% carbon dioxide (BOC, London, UK). The reactor was mixed with 0.5 volume of air per volume of liquid per minute (vvm) of 0.2 µm filtered air, and undertaken at 30 °C, under 100 µE·m^−2^·s^−1^ of artificial light, provided by two 18 W fluorescent bulbs (Grolux, BLT direct, Ipswich, UK).

#### 2.1.3. Testing the Parameter Space

A series of further investigations looking into the suitable parameter space within the 1 L reactors was also undertaken. This was achieved by fixing each of the parameters in turn and incrementally altering the others over a 7-day batch experiment. In the case of the temperature experimentation this was altered incrementally from 25 °C to 40 °C, while maintaining a mixing speed of 0.5 vvm. In the case of altering the mixing speed the temperature was held at 35 °C and the mixing speed was altered incrementally from 0.1 to 1 vvm. Finally, the effect of surface light irradiation on the initial growth rate was also investigated at a fixed temperature of 35 °C and aeration of 0.5 vvm.

#### 2.1.4. Nutrient Removal

Experiments investigating nutrient removal were undertaken at 35 °C, under 100 µE m^−2^ s^−1^ of artificial light, provided by two 18 W fluorescent bulbs (Gro-Lux) [18]. Mixing was induced by aerating the reactor with 0.5 vvm of 0.2 µm filtered air.

#### 2.1.5. Fed-Batch, and Concentration Effect Experimentation

Fed-batch and concentration experimentation was undertaken, using 1, 3 and 10 × BBM, and conditions outlined in Section 2.1.4. Fed-batch experimentation was compared to 3 × BBM concentration, with the feeding split into three 20 mL injections of BBM concentrate, equal a final concentration of 3 × BBM at time points 0, 48 and 72 h. 

### 2.2. Formulas

#### 2.2.1. Maximal Specific Growth Rate

The maximum specific growth rate ($\mu_{max}$) from the experiments was calculated according to Equation (1). Where $X_{1}$ and $X_{0}$ correspond to the algal density at times $t_{1}$ and $t_{0}$ respectively [27], and calculated using an average of the first 3 data points on the time series.

(1) $$\mu_{max}=\frac{\ln\left(X_{1}\right)-\ln\left(X_{0}\right)}{t_{1}-t_{0}}$$

#### 2.2.2. Final Yield and Productivity

The final biomass yield ($X_{Y}$) was determined by subtracting the final biomass concentration from the initial biomass concentration (Equation (2)). Biomass and lipid productivity ($P_{X}$ and $\;P_{L}$) were calculated on a batch basis (Equations (3) and (4)), by dividing the final product yield by the total number of hours or days within the experiment taken to reach stationary phase. Where $X_{1}$ and $X_{0}$ or $L_{1}$ and $L_{0}$ correspond to the algal density and lipid concentration at times $t_{1}$ and $t_{0}$ respectively [27].

(2) $$X_{Y}=X_{t}-X_{0}$$

(3) $$P_{X}=\frac{X_{1}-X_{0}}{t_{1}-t_{0}}$$

(4) $$P_{L}=\frac{L_{1}-L_{0}}{t_{1}-t_{0}}$$

#### 2.2.3. Doubling Time

The doubling time ($D_{t}$) was calculated according to the relationship described in Equation (5), using an appropriate specific growth rate (μ) [27].

(5) $$D_{t}=\frac{\ln\left(2\right)}{\mu }$$

#### 2.2.4. Substrate Uptake

Substrate uptake ($R_{s}$) was calculated on a batch basis (Equation (6)), by dividing the difference between the initial and final nutrient concentration by $S_{1}$ and $S_{0}$ which correspond to the nutrient concentrations at times $t_{1}$ and $t_{0}$ respectively [27].

(6) $$R_{s}=\frac{S_{1}-S_{0}}{t_{1}-t_{0}}$$

#### 2.2.5. Photosynthetic Yield on Photosynthetically Active Radiation (PAR)

The photosynthetic yield on PAR was calculated to provide a measure of the efficiency of photosynthesis within any given photobioreactor system and considers the relationship between yield ($X_{t}$), incident light ($PAR_{t}$) and surface area (A). The calculation is expressed in Equation (7) [28].

(7) $$Y_{PAR}=\frac{X_{t}}{A\;.\;PAR_{t}}$$

### 2.3. Biomass and Lipid Quantification Techniques

Each experimental condition was undertaken as a set of biological triplicate repeats, unless otherwise stated. Growth was monitored by measuring the optical density at 750 nm (CamSpec. Leeds, UK) [18] and converting it to a biomass dry weight. This was achieved by using a previously determined calibration curve. Actual dry weights were collected and concentrated by centrifugation (10 min at 4370 g), washed and lyophilized prior to weighing. Care was taken to prevent false readings by using the appropriate blank measurements and subtracting from those containing algae. Lipid accumulation was assessed by fluorescence spectroscopy using the fluorescent dye, Nile Red [29]. Staining was performed by adding Nile Red to culture samples to a final concentration of 2 µg/mL and allowing 150 seconds for the binding to occur. Fluorescence was measured using a Perkin-Elmer LS-55 Luminescence Spectrometer (Perkin-Elmer, Beaconsfield, UK) with the excitation wavelength set at 510 nm and the emission scanned between 530 and 750 nm, the emitted fluorescence from Nile Red bound to TAGs was recorded at 575–590 nm. Comparison to a Triolein standard (Sigma) was used for estimation of total lipid levels (see Appendix A).

### 2.4. Determining Nutrient and pH Levels

Ion chromatography (IC) was undertaken to analyze the nutrient uptake of *C. sorokiniana* in relation to reduction of nitrate, phosphate and sulfate levels. The samples were run on a KS-1100 IC instrument (Dionex, Thermo Fisher Scientific, Hemel Hempstead, UK), using an AS23 4 × 250 mm carbonate eluent anion-exchange column (Dionex). Anion mode analysis was carried out according to the manufacturer’s recommendations, using a mobile phase of 4.5 mM Na_2_CO_3_. The flow rate was set at 1 mL min^−1^, with a total run time of 30 min and temperature held at 30 °C. Cation analysis was undertaken using an IonPac CS16-5 µm (5 × 250 mm) column with 30 mM Methanesulfonic acid as the eluent (Thermo Fisher Scientific, Hemel Hempstead, UK). The flow rate was set at 1 mL·min^−1^, with a total run time of 25 min and temperature held at 40 °C. Detection of ion peaks in both conditions was undertaken by suppressed conductivity measurements at 25 mA. The spectra were analyzed using a set of standards and software provided by Dionex. The pH of the growth media was monitored over the course of the experiment with a pH probe (Mettler Toledo, Royston, UK).

### 2.5. Data Analysis

Data was analyzed and plotted on Windows Microsoft Excel 2010 (Microsoft, London, UK). Triplicate experimental results display error bars with 2 standard deviations from the mean. Significant differences between treatment conditions in Table 1 were analyzed by one-way ANOVA with a statistical significance of *p* ≤ 0.05.

## 3. Results

### 3.1. Growth under Different Carbon Sources

**Figure 2:**
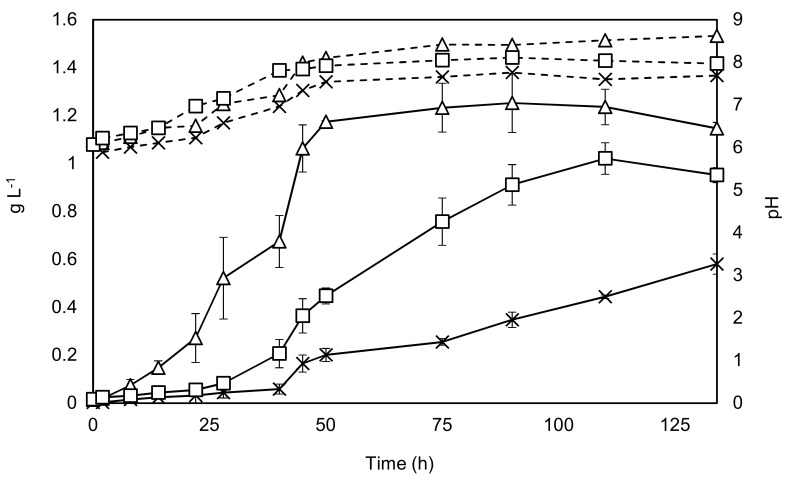
Growth of *C. sorokiniana* under phototrophic (with and without CO_2_ augmentation) and mixotrophic conditions. Solid black lines represent dry weight on the primary y-axis, while dashed lines represent pH which is shown on the secondary y-axis. Triangles—growth with 2 g·L^−1^ sodium acetate. Squares—growth with CO_2_ addition, Crosses—no addition of carbon source, except by mixing air. Experiments were undertaken in triplicate, and the error bars show 2 standard deviations from the mean.

The results from growing *C. sorokiniana* included growth both with and without the addition of an enriched CO_2_ stream, as well as growth with the addition of acetate. The results are shown in Figure 2.

Key parameters of the different growth conditions were determined and are displayed in Table 1.

Figure 2 and Table 1 show that all the key parameters improve considerably with the addition of sodium acetate, while the stationary phase can be reached in almost half of the time, findings supported by the literature [30,31]. The +/− CO_2_ experiments show that the maximal specific growth rate is unaffected by carbon dioxide addition at the beginning of the experiment. This is probably explained by the mixing aeration, which supplies sufficient carbon dioxide to dilute cultures with relatively low levels of biomass. However, as the culture grows denser the importance of carbon dioxide addition can be seen from 24 h onwards. Final yields were found to be almost 20% lower in the + CO_2_ condition when compared to the acetate condition, while the final yield was around 50% lower without carbon dioxide augmentation. These results indicate that augmentation with sodium acetate would be a promising bioprocessing option.

### 3.2. Testing the Parameter Space

**Figure 3:**
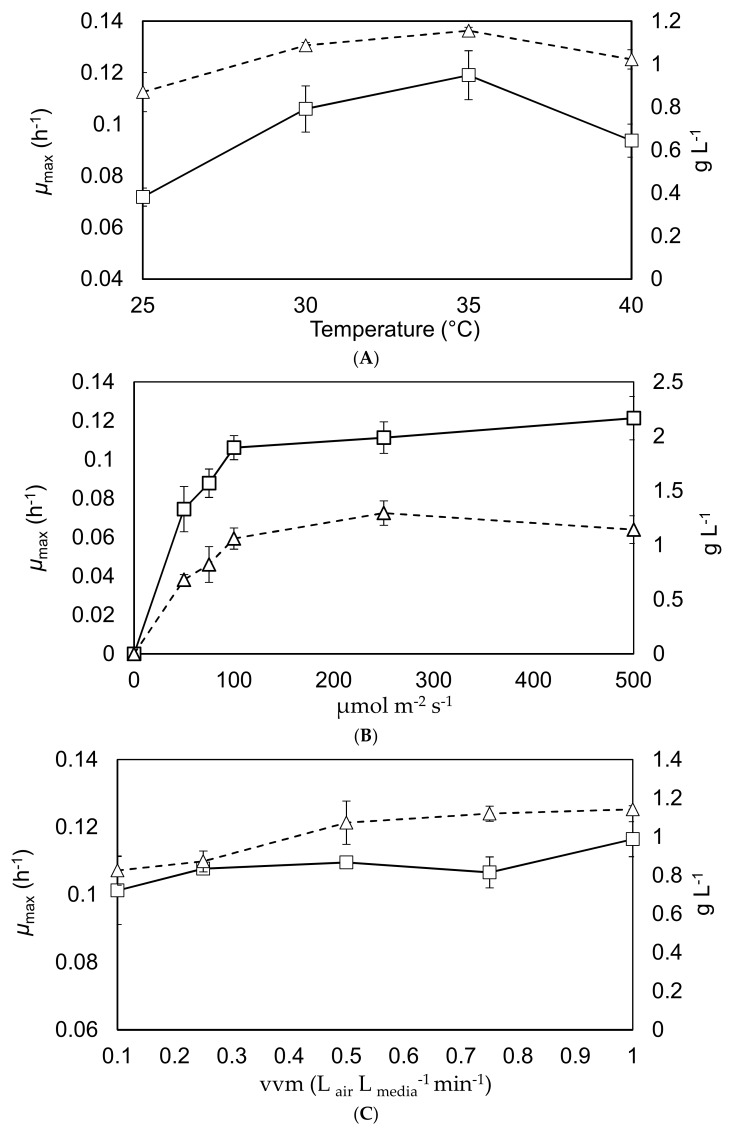
Maximum specific growth rates and final yields in the 1 L Duran bottle reactors. The primary y-axis shows the maximal growth rates (square markers, solid black lines), while the secondary y-axis shows the final yield after a 7-day batch (triangle marker, dashed black lines). Graph (**A**) demonstrates the effect of altering the temperature. Graph (**B**) indicates the response to changing the surface irradiance. Graph (**C**) demonstrates the effect caused by changing the agitation. Triplicate experiments, error bars show 2 standard deviations from the mean.

These experiments investigated how the maximal growth rate and final yield were affected by alteration of the temperature, mixing intensity and light intensity. The results are shown in Figure 3.

The results from Figure 3A shows that both the maximal growth rate and final yield increase with the temperature, to a maximum around 30–35 °C [12,31]. The results from Graph (B) are aligned with what would be expected within the literature in terms of maximum growth rate of *C. sorokiniana* under the tested conditions [32]; showing a maximum specific growth rate in the region of 0.12 h^−1^ at a surface irradiance between 100–500 µE·m^−2^·s^−1^. The results from Graph (C) show that mixing has a lower effect on the maximal growth rate and final yield than temperature and light intensity; although there is a slight increase in growth rate and yield as the vvm rises. Overall these results would suggest optimal operational conditions around 30–35 °C with surface irradiation of 100 µE·m^−2^·s^−1^ and an aeration rate above 0.2 vvm, which is generally supported by the literature [10,13,20].

### 3.3. Nutrient Removal and Lipid Production

**Figure 4:**
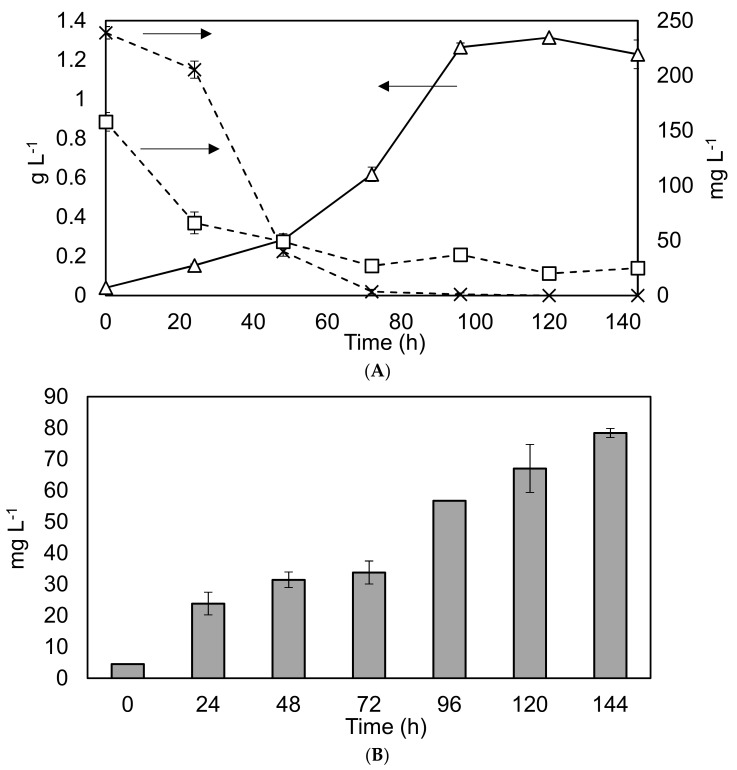
Nutrient removal and lipid production profiles in the 1 L Duran bottle reactor under different conditions. Graph (**A**) Solid black line with triangles represents biomass dry weight on the primary y-axis, while the dashed lines represent nutrient depletion on the secondary y-axis. Squares: phosphate levels. Crosses: nitrate levels. Experiments were undertaken in triplicate, and the error bars show one standard deviation from the mean, arrows clarify the axis to which the data corresponds. Graph (**B**) Grey bars show the lipid concentration. Triplicate experiments, error bars show 2 standard deviations.

As part of scoping the potential for nutrient removal and lipid production within larger scale operations, *C. sorokiniana* was grown within 1 L Duran Bottles using BBM. The results are shown in Figure 4.

The findings confirm reports within the literature of rapid growth and nutrient removal rates under similar conditions [12]. The high removal rates of nitrogen and phosphorus under controlled laboratory conditions (37 and 30 mg·L^−1^·d^−1^ respectively), would indicate considerable potential for wastewater remediation. However, the relatively low average lipid productivity of 9.2 mg·L^−1^·d^−1^, (corresponding to 4.5–16% of DW) within the biomass means that UTEX 1230 may not be well suited to biodiesel production.

### 3.4. Fed-Batch and Concentration Effects

**Figure 5:**
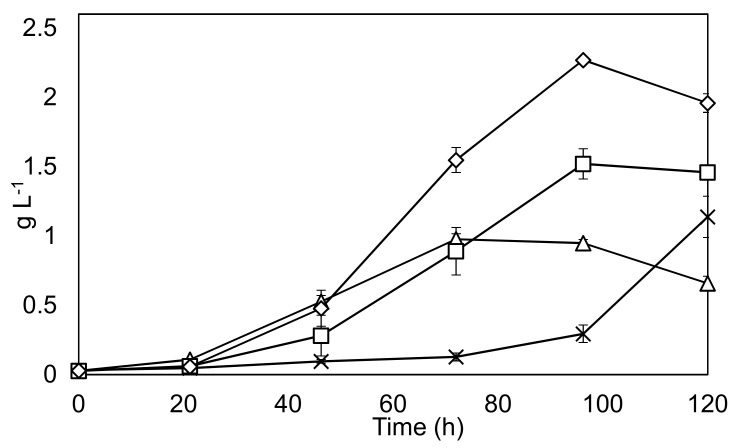
Optimization of feeding strategy. Solid black lines represent biomass dry weight on y-axis. Triangles: 1 × BBM, Squares: 3 × BBM, Diamonds: 3 × BBM fed batch, Crosses 10 × BBM. Experiments were undertaken in triplicate, and the error bars show 2 standard deviations from the mean.

An exploration of the best growth strategy in terms of BBM concentration and feeding schedule was undertaken. The results are shown in Figure 5.

The findings show that a fed-batch strategy can increase the final yield by around 50% compared to a conventional batch run. Productivity is also considerably improved between 48–96 h in the fed-batch condition. Also, of interest is the fact that *C. sorokiniana* appears to be able to tolerate the very high nutrient concentrations found in 10 × BBM, albeit with a considerably reduced growth rate over the course of the experimentation.

## 4. Discussion

The findings within this research paper show that *Chlorella sorokiniana* UTEX 1230 is well suited to small scale work within laboratory bubble columns, although more research is required to ascertain performance at larger scales. Key findings within this paper indicate that *C. sorokiniana* displays a maximal growth rate in the region of 0.12 h^−1^, as well as averaged batch productivity under conditions of continuous illumination outlined in this paper in the region of 0.22–0.38 g L^−1^·d^−1^. These findings fall within a range supported by the literature given the experimental conditions [19,33] and are at the higher end of many other phototrophically grown algal strains under these conditions [34,35]. In fact, a comparison to other studies shows the maximal growth rate of *C. sorokiniana* found within these experiments is higher than other species of *Chlorella* [36], and similar to other sub-species of *Chlorella sorokiniana* (0.11–0.16 h^−1^) [13]. The low levels of lipid production confirms previous findings at around 10–25% of DW [31,37], meaning *C. sorokiniana* UTEX 1230 may not be well suited to biodiesel production, especially when compared to other *Chlorella* species, which are capable of producing up to 0.33 g·L^−1^·d^−1^ of lipid [38]. Addition of acetate had a significant impact on the maximal specific growth rate, productivity, yield and doubling times. The effects in this regard are similar to literature values showing growth on glucose [15], which show up to 5 × increase in growth rates for UTEX 1230 under heterotrophic conditions compared to phototrophic cultivation [39]. The findings also show that high nutrient removal rates are achievable (close to 100% for NO_3_, and 83% for PO_4_), comparable to previous studies [19] and indicating potential suitability for wastewater treatment. However, it was found that higher nutrient concentrations decreased the specific growth rates, which could have major implications for wastewater treatment and other bioprocesses. This can be explained by the unfavorable conditions brought on by high nutrient environments, resulting from increased osmotic stress and intermediate inhibition [40]. This analysis is further supported by data from the batch experiments, which show that maximal averaged growth rates, productivities and yields could be obtained under a staggered feeding regime. As a result, the study demonstrates that the use of a fed-batch approach to nutrient loading can improve the rate of total nutrient removal, while simultaneously increasing the microalgal productivity and yield. 

## 5. Conclusions

The findings outlined within this research paper show that *Chlorella sorokiniana* UTEX 1230 is a robust alga, well suited to cultivation within bubble column-type photobioreactors. The strain displays many favorable characteristics, including rapid growth rates under a range of conditions and high levels of nutrient removal. Points of interest include the doubling of productivity brought on by the addition of acetate, as well as improved growth rates resulting from a fed-batch mode of inorganic nutrient loading. In combination these attributes make the strain an attractive target for industrial biotechnology or wastewater treatment, where high productivity and nutrient removal rates are desirable. Future work should aim to replicate these findings at a larger scale, while also exploring an improved fed-batch approach; which could for example investigate the addition of multiple acetate and inorganic nutrient loadings during the cultivation. Furthermore, the extrapolation of these acetate feeding and fed-batch approaches into higher-value algae strains may yield particularly interesting improvements in bioproduction.

## Figures and Tables

**Table 1:** Biological parameters under differing growth conditions. A one-way independent ANOVA was performed to compare whether values in different treatment groups we significantly different from one another. Values marked with * indicate significance between sodium acetate treatment and +/− carbon dioxide treatments. While values marked with ^ indicate a significant difference between + carbon dioxide against—carbon dioxide (*p* ≤ 0.05).

Carbon Source	$\mu_{max}$ (h^−1^)	$\;X_{Y}$ (g·L^−1^)	$\;P_{X}$ (g·L^−1^·d^−1^)	$\;D_{t}$ (h^−1^)
Sium acetate	0.21 *	1.25 *	0.6 *	3.3 *
+ Carbon dioxide	0.102	1.01 ^	0.22 ^	6.8
− Carbon dioxide	0.107	0.58	0.1	6.5

**Figure A1:**
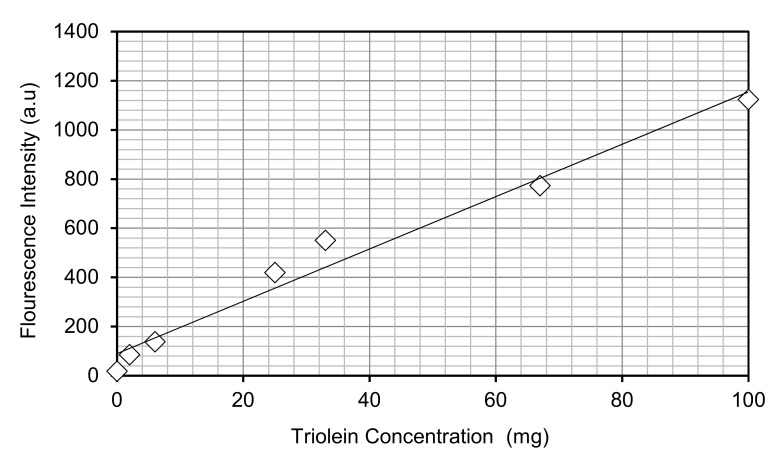
Relationship between triolein concentration and fluorescence intensity.

## References

[1] Ebenezer V., Medlin L.K., Ki J.S. (2012). Molecular Detection, Quantification, and Diversity Evaluation of Microalgae. Mar. Biotechnol..

[2] Borowitzka M. (1992). Algal biotechnology products and processes—Matching science and economics. J. Appl. Phycol..

[3] Wu H.L., Hseu R.S., Lin L.P. (2001). Identification of *Chlorella* spp. isolates using ribosomal DNA sequences. Bot. Bull. Acad. Sin..

[4] Furnas M.J. (1990). Insitu growth-rates of marine-phytoplankton—Approaches to measurement, community and species growth-rates. J. Plankton Res..

[5] Sorokin C., Myers J. (1953). A high-temperature strain of *Chlorella*. Science.

[6] Kunz W.F. (1972). Response of the alga *Chlorella sorokiniana* to 60 Co gamma radiation. Nature.

[7] Kessler E. (1985). Upper limits of temperature for growth in *Chlorella* (Chlorophyceae). Plant Syst. Evolut..

[8] Dorr R., Huss V.A.R. (1990). Characterization of nuclear-DNA in 12 species of *Chlorella* (Chlorococcales, Chlorophyta) by DNA reassociation. Biosystems.

[9] Kessler E., Huss V.A.R. (1992). Comparative physiology and biochemistry and taxonomic assignment of the *Chlorella* (Chlorophyceae) strains of the culture collection of the university of texas at ausin. J. Phycol..

[10] Ramanna L., Guldhe A., Rawat I., Bux F. (2014). The optimization of biomass and lipid yields of *Chlorella sorokiniana* when using wastewater supplemented with different nitrogen sources. Bioresour. Technol..

[11] Bohutskyi P., Kligerman D.C., Byers N., Nasr L.K., Cua C., Chow S., Su C., Tang Y., Betenbaugh M.J., Bouwer E.J. (2016). Effects of inoculum size, light intensity, and dose of anaerobic digestion centrate on growth and productivity of *Chlorella* and *Scenedesmus* microalgae and their poly-culture in primary and secondary wastewater. Algal Res..

[12] De-Bashan L.E., Trejo A., Huss V.A.R., Hernandez J.P., Bashan Y. (2008). Chlorella sorokiniana UTEX 2805, a heat and intense, sunlight-tolerant microalga with potential for removing ammonium from wastewater. Bioresour. Technol..

[13] Janssen M., Kuijpers T.C., Veldhoen B., Ternbach M.B., Tramper J., Mur L.R., Wijffels R.H. (1999). Specific growth rate of *Chlamydomonas reinhardtii* and *Chlorella sorokiniana* under medium duration light/dark cycles: 13–87 s. J. Biotechnol..

[14] Wan M.X., Wang R.M., Xia J.L., Rosenberg J.N., Nie Z.Y., Kobayashi N., Oyler G.A., Betenbaugh M.J. (2012). Physiological evaluation of a new *Chlorella sorokiniana* isolate for its biomass production and lipid accumulation in photoautotrophic and heterotrophic cultures. Biotechnol. Bioeng..

[15] Lee Y.K., Ding S.Y., Hoe C.H., Low C.S. (1996). Mixotrophic growth of *Chlorella sorokiniana* in outdoor enclosed photobioreactor. J. Appl. Phycol..

[16] Lane C.D., Coury D.A., Allnutt F.C.T. (2017). Composition and Potential Products from *Auxenochlorella protothecoides*, *Chlorella sorokiniana* and *Chlorella vulgaris*. Ind. Biotechnol..

[17] Béchet Q., Muñoz R., Shilton A., Guieysse B. (2012). Outdoor cultivation of temperature-tolerant *Chlorella sorokiniana* in a column photobioreactor under low power-input. Biotechnol. Bioeng..

[18] Lizzul A., Hellier P., Purton S., Baganz F., Ladommatos N., Campos L. (2014). Combined remediation and lipid production using *Chlorella sorokiniana* grown on wastewater and exhaust gases. Bioresour. Technol..

[19] Bohutskyi P., Liu K., Nasr L.K., Byers N., Rosenberg J.N., Oyler G.A., Betenbaugh M.J., Bouwer E.J. (2015). Bioprospecting of microalgae for integrated biomass production and phytoremediation of unsterilized wastewater and anaerobic digestion centrate. Appl. Microbiol. Biotechnol..

[20] Belkoura M., Benider A., Dauta A. (1997). Effects of temperature, light intensity and growth phase on the biochemical composition of *Chlorella sorokiniana* Shihira & Krauss. Ann. Limnol. Int. J. Limnol..

[21] Illman A.M., Scragg A.H., Shales S.W. (2000). Increase in *Chlorella* strains calorific values when grown in low nitrogen medium. Enzyme Microb. Technol..

[22] Gouveia L., Oliveira A.C. (2009). Microalgae as a raw material for biofuels production. J. Ind. Microbiol. Biotechnol..

[23] Kumar K., Dasgupta C.N., Nayak B., Lindblad P., Das D. (2011). Development of suitable photobioreactors for CO_2_ sequestration addressing global warming using green algae and cyanobacteria. Bioresour. Technol..

[24] Mizuno Y., Sato A., Watanabe K., Hirata A., Takeshita T., Ota S., Sato N., Zachleder V., Tsuzuki M., Kawano S. (2013). Sequential accumulation of starch and lipid induced by sulfur deficiency in *Chlorella* and *Parachlorella* species. Bioresour. Technol..

[25] Matsukawa R., Hotta M., Masuda Y., Chihara M., Karube I. (2000). Antioxidants from carbon dioxide fixing *Chlorella sorokiniana*. J. Appl. Phycol..

[26] Dawson H.N., Burlingame R., Cannons A.C. (1997). Stable Transformation of *Chlorella*: Rescue of Nitrate Reductase-Deficient Mutants with the Nitrate Reductase Gene. Curr. Microbiol..

[27] Doran P.M. (1995). Bioprocess Engineering Principles.

[28] Franco M.C., Buffing M.F., Janssen M., Lobato C.V., Wijffels R.H. (2012). Performance of *Chlorella sorokiniana* under simulated extreme winter conditions. J. Appl. Phycol..

[29] Cooksey K.E., Guckert J.B., Williams S.A., Callis P.R. (1987). Fluorometric determination of the neutral lipid content of microalgal cells using Nile Red. J. Microbiol. Methods.

[30] Wan M., Liu P., Xia J., Rosenberg J.N., Oyler G.A., Betenbaugh M.J., Nie Z., Qiu G. (2011). The effect of mixotrophy on microalgal growth, lipid content, and expression levels of three pathway genes in *Chlorella sorokiniana*. Appl. Microbiol. Biotechnol..

[31] Vonlanthen S. (2013). Analysis and Manipulation of Storage Lipids in Microalgae. Ph.D. Thesis.

[32] Sorokin C., Krauss R.W. (1958). The Effects of Light Intensity on the Growth Rates of Green Algae. Plant Physiol..

[33] Sorokin C., Krauss R.W. (1959). Maximum growth rates of chlorella in steady-state and in synchronized cultures. Proc. Natl. Acad. Sci. USA.

[34] Ugwu C.U., Aoyagi H., Uchiyama H. (2008). Photobioreactors for mass cultivation of algae. Bioresour. Technol..

[35] Fernández F.G.A., Sevilla J.M.F., Pérez J.A.S., Grima E.M., Chisti Y. (2001). Airlift-driven external-loop tubular photobioreactors for outdoor production of microalgae: Assessment of design and performance. Chem. Eng. Sci..

[36] Wang L., Min M., Li Y., Chen P., Chen Y., Liu Y., Wang Y., Ruan R. (2010). Cultivation of Green Algae *Chlorella* sp. in Different Wastewaters from Municipal Wastewater Treatment Plant. Appl. Biochem. Biotechnol..

[37] Kobayashi N., Noel E.A., Barnes A., Watson A., Rosenberg J.N., Erickson G., Oyler G.A. (2013). Characterization of three *Chlorella sorokiniana* strains in anaerobic digested effluent from cattle manure. Bioresour. Technol..

[38] Přibyl P., Cepák V., Zachleder V. (2012). Production of lipids in 10 strains of *Chlorella* and *Parachlorella*, and enhanced lipid productivity in *Chlorella vulgaris*. Appl. Microbiol. Biotechnol..

[39] Rosenberg J.N., Kobayashi N., Barnes A., Noel E.A., Betenbaugh M.J., Oyler G.A. (2014). Comparative Analyses of Three *Chlorella* Species in Response to Light and Sugar Reveal Distinctive Lipid Accumulation Patterns in the Microalga *C. sorokiniana*. PLoS ONE.

[40] Gilmour D.J., Hipkins M.F., Boney A.D. (1984). The effect of osmotic and ionic stress on the primary processes of photosynthesis in *Dunaliella tertiolecta*. J. Exp. Bot..

